# Acknowledgments to Reviewers 2023

**DOI:** 10.1093/gpbjnl/qzae038

**Published:** 2024-06-11

The editorial team of GPB wishes to extend a heartfelt thanks to the GPB reviewers who have provided valuable comments for manuscripts under consideration by GPB during 2023. Your contributions are vital for the ever-improving scientific quality of the journal. From 2024 onward, GPB is published by the Oxford University Press. We look forward to your continued support in 2024 and the years to come!

**Table:** 

Kami Ahmad	*USA*
Shahid Akbar	*Pakistan*
Klaus-Jürgen Appenroth	*Germany*
Zhiliang Bai	*USA*
Weining Bai	*China*
Angela S. Baker	*USA*
Yiming Bao	*China*
Anna Bernasconi	*Italy*
Dongbo Bu	*China*
Jing Cai	*China*
Yudong Cai	*China*
Lijun Cao	*China*
Stefano Ceri	*Italy*
Yingguang Chan	*Germany*
Chen Chen	*China*
Kaifu Chen	*USA*
Yamei Chen	*USA*
Yiyun Chen	*USA*
Yue Chen	*USA*
Zhanheng Chen	*China*
Zhen Chen	*China*
Zhong-Hua Chen	*Australia*
Kunqi Chen	*China*
Lianmin Chen	*China*
Ming Chen	*China*
Qipian Chen	*China*
Shengquan Chen	*China*
Weihua Chen	*China*
Xing Chen	*China*
Di Chen	*China*
Fei Chen	*China*
Hailin Chen	*China*
Hebing Chen	*China*
Hua Chen	*China*
Changde Cheng	*USA*
Feng Cheng	*China*
Han Cheng	*China*
Hong Cheng	*China*
Jinke Cheng	*China*
Andrea Maria Chiariello	*Italy*
Guy R. Cochrane	*UK*
Lindsay Grey Cowell	*USA*
Qinghua Cui	*China*
Jie Cui	*China*
Peng Cui	*China*
Xuefeng Cui	*China*
Rachel Culp-Hill	*USA*
Qi Dai	*China*
Michiel de Hoon	*Japan*
Abdollah Dehzangi	*USA*
Lei Deng	*China*
Xinxian Deng	*USA*
Zhana Duren	*USA*
Fatma-Elzahraa Eid	*USA*
Jesse Engreitz	*USA*
Hang Fan	*China*
Hengyu Fan	*China*
Xiaohui Fan	*China*
Yan Fu	*China*
Monika Fuxreiter	*Italy*
Fei Gao	*China*
Fei Gao	*China*
Lei Gao	*China*
Zhancheng Gao	*China*
Jing Gong	*China*
Michael Gromiha	*India*
Jin Gu	*China*
Lianfeng Gu	*China*
Yunyan Gu	*China*
An-yuan Guo	*China*
Weilong Guo	*China*
Guoji Guo	*China*
Li Guo	*China*
Wenbo Guo	*China*
Jianyong Han	*China*
Jing-Dong J. Han	*China*
Leng Han	*USA*
Yan Hao	*China*
Shunmin He	*China*
Aibin He	*China*
Chunjiang He	*China*
Chunhui Hou	*China*
Tingjun Hou	*China*
Shixian Hu	*China*
August Yue Huang	*USA*
Guangping Huang	*China*
Hsien-Da Huang	*China*
Jinyan Huang	*China*
Lin Huang	*China*
Sanwen Huang	*China*
Tao Huang	*China*
Mark Izraelson	*Russian Federation*
Peng Ji	*USA*
Zhicheng Ji	*USA*
Peilin Jia	*China*
Xinmiao Jia	*China*
Haifeng Jiang	*China*
Ning Jiang	*USA*
Rui Jiang	*China*
Wei Jiang	*China*
Yu Jiang	*China*
Yuannian Jiao	*China*
Craig T. Jordan	*USA*
Yinglei Lai	*USA*
Sangkyu Lee	*Republic of Korea*
Haikuo Li	*USA*
Leyuan Li	*China*
Yu Li	*China*
Yueying Li	*China*
Zhengqiu Li	*China*
Mo Li	*China*
Shuai Cheng Li	*Hong Kong, China*
Shuwei Li	*China*
Tingting Li	*China*
Wei Li	*China*
Wei Li	*China*
Xiang Li	*China*
Xiaoyu Li	*China*
Yonghui Li	*USA*
Dong Li	*China*
Fuhai Li	*USA*
Guohui Li	*China*
Qi Liao	*China*
Lan Lin	*USA*
Feng Liu	*China*
Qi Liu	*China*
Ru-Juan Liu	*China*
Shiyong Liu	*China*
Xingyin Liu	*China*
Yong-Xin Liu	*China*
Ze-Xian Liu	*China*
Yu Liu	*China*
Yuan Liu	*USA*
Wanlu Liu	*China*
Xiaolei Liu	*China*
George E. Liu	*USA*
Jianxiao Liu	*China*
Jun Liu	*China*
Jun Liu	*China*
Lin Liu	*China*
Anne Lopes	*France*
Jian Lu	*China*
Mei Luo	*USA*
Haitao Luo	*China*
Lina Ma	*China*
Jia Meng	*China*
Zhichao Miao	*China*
Wenwen Min	*China*
Hossein Moosaei	*Czech Republic*
Jason B. Muhitch	*USA*
Eran A. Mukamel	*USA*
Zemin Ning	*UK*
Yamei Niu	*China*
Shujiro Okuda	*Japan*
Jianbo Pan	*China*
Yanli Pang	*China*
Elahe Parvizi	*New Zealand*
Gita Pathak	*USA*
Georgios Pavlopoulos	*Greece*
Jiajie Peng	*China*
Guangdun Peng	*China*
Dylan Plummer	*USA*
John R. Prensner	*USA*
Fred W. Prior	*USA*
Jiang Qian	*USA*
Liang Qiao	*China*
Qiang Qiu	*China*
Anthony Redmond	*Ireland*
Xianwen Ren	*China*
Yan Ren	*China*
Aiming Ren	*China*
Jian Ren	*China*
Paul F. Robbins	*USA*
Xavier Robin	*Switzerland*
Mohamad Roshanzamir	*Islamic Republic of Iran*
Jue Ruan	*China*
Hang Ruan	*China*
Jorge Ruiz-Orera	*Germany*
Sudipto Saha	*India*
Chenghua Shao	*USA*
Feng Shao	*China*
Zhen Shao	*China*
Hong-Bin Shen	*China*
Hui Shen	*USA*
Xiaopei Shen	*China*
Junpeng Shi	*China*
Lihong Shi	*China*
Letitia Sng	*Australia*
Johannes Söding	*Germany*
Jiangning Song	*Australia*
Li Song	*USA*
Shuhui Song	*China*
Xiaofeng Song	*China*
Martin Steinegger	*Republic of Korea*
Julian Stingele	*Germany*
Jianzhong Su	*China*
Yapeng Su	*USA*
Zhen Su	*China*
Shiwei Sun	*China*
Lei Sun	*China*
Shisheng Sun	*China*
Minjia Tan	*China*
Yukun Tan	*USA*
Chris Soon Heng Tan	*China*
Haixu Tang	*USA*
Jing Tang	*Finland*
Bo Tang	*China*
Sheng-ce Tao	*China*
Andrew Teschendorff	*China*
Vijay Tiwari	*UK*
Tomotaka Ugai	*USA*
Cuihong Wan	*China*
Kun Wang	*China*
Xiu-Jie Wang	*China*
William Wang	*UK*
Xiyin Wang	*China*
Kai Wang	*USA*
Jianmin Wang	*USA*
Dong Wang	*China*
Fang Wang	*China*
Fengping Wang	*China*
Guodong Wang	*China*
Hongsheng Wang	*China*
Jiebiao Wang	*USA*
Jiguang Wang	*Hong Kong, China*
Juexin Wang	*USA*
Lu Wang	*China*
Minxian Wang	*China*
Shi Wang	*China*
Wen Wang	*China*
Bo Wang	*China*
Chenfei Wang	*China*
Yijie Wang	*USA*
Xiaolong Wang	*China*
Xiaotao Wang	*China*
Cynthia Webster	*USA*
Lei Wei	*USA*
Jia Wen	*USA*
Lu Wen	*China*
Aiping Wu	*China*
Zhiqiang Wu	*China*
Hao Wu	*USA*
Qiang Wu	*China*
Wei Wu	*China*
Xuebing Wu	*USA*
Lihong Xiao	*China*
Yi Xiao	*China*
Yutao Xiao	*China*
Yubin Xie	*China*
Zhengwei Xie	*China*
Yi Xiong	*China*
Bingxiang Xu	*China*
Dong Xu	*USA*
Juan Xu	*China*
Lingyang Xu	*China*
Peng Xu	*China*
Yungang Xu	*China*
Liangjiao Xue	*China*
Yu Xue	*China*
Fangrong Yan	*China*
Jun Yan	*China*
Ying Yang	*China*
Jian Yang	*China*
Can Yang	*Hong Kong, China*
Jian-Rong Yang	*China*
Tingting Yang	*China*
Xuerui Yang	*China*
Hui Ye	*China*
Keqiong Ye	*China*
Mingliang Ye	*China*
Xinhai Ye	*China*
Chengqi Yi	*China*
Jasmine Y. Young	*USA*
Jun Yu	*China*
Jiyang Yu	*USA*
Bin Yu	*China*
Guangchuang Yu	*China*
Li Yu	*China*
Miao Yu	*China*
Zhiyuan Yuan	*China*
Feng Yue	*USA*
Chongzhi Zang	*USA*
Zexian Zeng	*China*
Jixian Zhai	*China*
Shihua Zhang	*China*
Zhao Zhang	*China*
Yuanwei Zhang	*China*
Jing Zhang	*USA*
Kunlin Zhang	*China*
Rui Zhang	*China*
Rui Zhang	*China*
Xingtan Zhang	*China*
Zhihua Zhang	*China*
Zhiyan Zhang	*China*
Zhuqing Zhang	*China*
Ziding Zhang	*China*
Yifan Zhang	*China*
Yong Zhang	*China*
Yong Zhang	*China*
Tongwu Zhang	*USA*
Xiangmin Zhang	*China*
Xiao-Bing Zhang	*China*
Lei Zhang	*China*
Qiangfeng Zhang	*China*
Baohong Zhang	*USA*
Chi Zhang	*USA*
Zhigang Zhang	*China*
Yi Zhao	*China*
Fangqing Zhao	*China*
Guoyan Zhao	*USA*
Min Zhao	*Australia*
Shuhong Zhao	*China*
Zheng Zhao	*China*
Caihong Zheng	*China*
Meizhen Zheng	*China*
Mingyue Zheng	*China*
Yaoqi Zhou	*China*
Yu Zhou	*China*
Yue Zhou	*China*
Chenxi Zhou	*UK*
Fengfeng Zhou	*China*
Hu Zhou	*China*
Keren Zhou	*USA*
Qian Zhou	*China*
Wanding Zhou	*USA*
Ping Zhu	*China*
Xiaoyu Zhu	*China*
Yunping Zhu	*China*
Quan Zou	*China*
Zhixiang Zuo	*China*

